# Unusual Presentation of an Orphan Syndrome Masquerading as Castleman Disease in a Young Adult: Diagnostic Challenges and Clinical Implications: A Case Report

**DOI:** 10.1002/ccr3.70224

**Published:** 2025-03-27

**Authors:** Mohamed Gaafar Mohamedali, Bassem Alhariri, Sana Mohamed, Osman Osama Elhassan, Muayad Ahmad, Afraa Fadul

**Affiliations:** ^1^ Department of Medicine Hamad Medical Corporation, HMGH Doha Qatar; ^2^ Weill Cornell Medicine Doha Qatar; ^3^ Qatar University Medicine College Doha Qatar; ^4^ Medical Education Department Hamad Medical Corporation Doha Qatar

**Keywords:** Castleman disease, differential diagnosis, groin swelling, histopathology, IgG4‐related disease, lymphadenopathy

## Abstract

IgG4‐related disease (IgG4‐RD) is a rare and diagnostically challenging fibroinflammatory condition characterized by diverse clinical presentations. We present the case of a 33‐year‐old male with a progressively enlarging right groin swelling over 6 months. A similar episode occurred 4 years prior in Nepal, with an inconclusive biopsy. Initial investigations pointed to chronic subcutaneous infectious granulomatous disease with lymphadenopathy. Following multiple missed appointments, the patient underwent excision of a soft tissue mass and lymph nodes. Histopathology revealed features consistent with IgG4‐RD, including follicular hyperplasia and a Castleman disease‐like pattern, negative for malignancy. This case underscores the diagnostic challenges of IgG4‐RD and the importance of considering it in the differential diagnosis of chronic lymphadenopathy. Timely follow‐up, histological evaluation, and awareness of IgG4‐RD diagnostic criteria are crucial for proper management and avoiding delayed treatment.

**Summary**

- Diagnosing IgG4‐RD requires meticulous evaluation of clinical, radiological, and histopathological data, as it mimics conditions like Castleman disease and sarcoidosis.
- Heightened awareness of IgG4‐RD diagnostic criteria, including organ‐specific IgG4+ plasma cell counts and serum IgG4 levels, is crucial for timely intervention and prevention of irreversible organ damage.

**Abbreviations**
CD: Castleman disease
CT: computed tomography
HSCT: hematopoietic stem cell transplantation
MCD: multicentric Castleman's
MDT: multidisciplinary team
MRI: magnetic resonance imaging
MV: mixed variation
RD: related diseases
UCD: unicentric
US: ultrasound

## 1 Introduction

IgG4‐related disease (IgG4‐RD) is a systemic immune‐mediated fibroinflammatory disorder characterized by elevated serum IgG4 levels, lymphoplasmacytic infiltration, and storiform fibrosis. Its diverse manifestations and overlap with other diseases, including Castleman disease (CD), pose diagnostic challenges. This case highlights the importance of considering IgG4‐RD in the differential diagnosis of chronic lymphadenopathy and underscores the value of histopathological analysis in reaching an accurate diagnosis.

## 2 Case Presentation

### 2.1 Patient History

A 33‐year‐old gentleman with an unremarkable medical history presented to the hospital in November 2023 with a six‐month history of progressively enlarging right groin swelling. Notably, he had experienced a similar episode of right groin swelling 5 years prior, which had been evaluated with inconclusive biopsy results during his stay in Nepal.

### 2.2 Presenting Symptoms

The patient denied any constitutional symptoms such as fever, night sweats, or unexplained weight loss. On examination, a palpable right inguinal mass was noted without associated skin changes or tenderness.

### 2.3 Laboratory Findings

Laboratory investigations demonstrated a mild elevation in total bilirubin (4 mg/dL) Table 1, and total protein (81 g/L) Table 1, with no abnormalities in white blood cell count, hemoglobin, platelets, or renal function tests. Serum IgG4 levels were unfortunately not available for analysis.

**TABLE 1:** Laboratory investigation.

Parameters	2023	2024	Reference values
Total leukocytes	15.6	13.4	(4.0–10.0 × 10^3^/uL)
Platelet	178	155	(150–410 × 10^3^/uL)
Hemoglobin	12.6	11.7	(13–17 g/dL)
Absolute neutrophil manual (ANC)	14.5	10.8	(2.0–7.0)
Hematocrit	36.7	345	(40%–50%)
Prothrombin time (seconds)	11.6	12.5	(9.4–12.5)
International normalized ratio (INR)	1	1.1	Less than 1.2
Activated partial thromboplastin time (APTI) (seconds)	30.4	32.2	(25.1–36.3 s)
C reactive protein (mg/L)	116.7	126	(0–5)
Serum potassium K (mmol/L)	4.5	4.6	(3.5–5.3)
Serum sodium (mmol/L)	132	139	(133–146)
Serum chloride (mmol/L)	95	102	(95–108)
Serum calcium (mmol/L)	2.51	2.41	(0.7–1)
Serum urea (mmol/L)	6.8	2.7	(2.5–7.8)
Serum creatinine (umol/L)	89	64	(62–106)
Serum glucose (mmol/L)	6.4	5.6	(3.3–5.5)
Serum albumin (gm/L)	47	41	(35–50)
Serum total protein (gm/L)	90	76	(60–80)
Lactate (mmol/L)		1.9	(0.5–2.2)
AST (IU/L)	14	11	(0–41)
ALT (IU/L)	16	12	(0–41)
Alkaline phosphatase (U/L)	59	54	(40–129)
Serum total bilirubin (mg/dL)	23	4	(0–20)
Hepatitis Serology			

### 2.4 Diagnostic Workup

**FIGURE 1:**
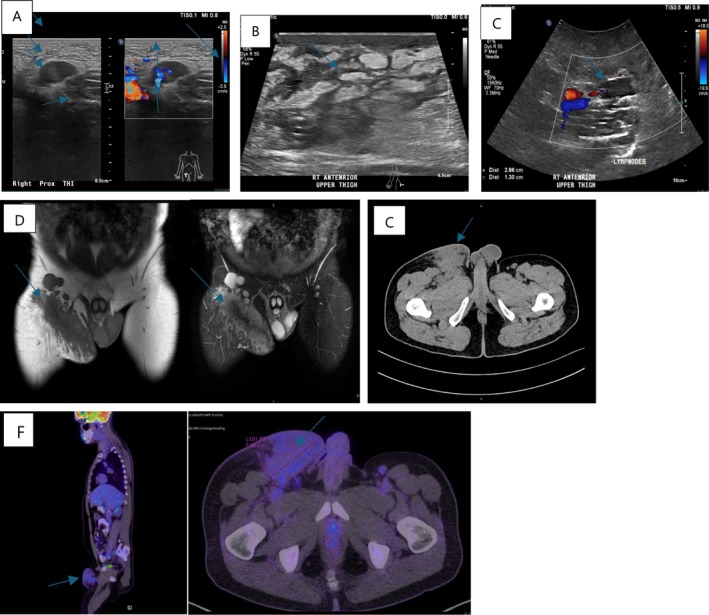
(A) US 2021 Rt anterior upper thigh with doppler. (B) US 2023 Rt anterior upper thigh. (C) US 2023 Rt anterior upper thigh with doppler. (D) MRI, coronal section with and without contrast. (D, E) right thigh raised suspicion for chronic subcutaneous infection. Granulomatous disease with associated lymphadenopathy (blue arrows). (E) CT pelvis, transverse section. (F) PET scan, right upper anterior thigh suggestive of an inflammatory process with low activity, likely infected/inflammatory lymph nodes and reactive single right axillary and left inguinal lymph nodes. (blue arrows).

US Groin and inguinal region in 2021 showed findings suggestive of a soft tissue mass (Figure 1A), US was repeated again in November 2023 and showed multiple enlarged lymph nodes(Figure 1B,C) CT Scan of the pelvis showed findings suggestive of a subcutaneous soft tissue enhancing lesion and multiple inguinal and external iliac lymph node enlargement suggesting an inflammatory process (Figure 1E). Magnetic resonance imaging (MRI) of the right thigh raised suspicion for chronic subcutaneous infectious granulomatous disease with associated lymphadenopathy (Figure 1D). Despite being scheduled for a biopsy several times and several missed appointments, the patient eventually underwent excision of the right inguinal soft tissue mass and lymph node on March 11, 2024. PET Scan was done and showed findings in the right upper anterior thigh suggestive of an inflammatory process with low activity, likely infected/inflammatory lymph nodes and reactive single right axillary and left inguinal lymph nodes.

#### 2.4.1 Flow Cytometry Analysis

On right upper thigh mass showed approximately 66% T‐cells expressing CD3, CD5, and CD7 (majority) with CD4:CD8 ratio at approximately 4.5:1. There are approximately 17% B‐cells expressing CD19 and CD20 with kappa: lambda ratio at approximately 1.6:1. There is a subpopulation of B‐cells (approximately 2%) showing brighter expression of CD20 and expressing CD10, which may represent germinal center B‐cells. There is no definitive immunophenotypic evidence of a monotypic B‐cell population. CD45‐negative events comprise approximately 4%.

### 2.5 Histopathology

Examination of the excised tissue revealed prominent lymphoid hyperplasia within the lymph nodes and soft tissue, numerous eosinophils, sclerosis, and fibrosis in the soft tissue. These features were consistent with IgG4‐related disease exhibiting a follicular hyperplasia/CD‐like pattern. Malignancy was definitively excluded.

### 2.6 Treatment and Outcome

The patient did not receive any new treatment yet, as he is asymptomatic for now. The MDT meeting was done, and it was advised to conduct hematological surveillance and to perform endoscopic procedures, and to refer the patient to Rheumatology for investigation and observation from their side for now.

## 3 Discussion

Fibro‐inflammatory diseases such as IgG4‐related disease can impact almost any organ system. Major enlargement of the salivary and lacrimal glands, ocular disease, autoimmune pancreatitis, retroperitoneal fibrosis, and tubulointerstitial nephritis are among the common manifestations [1].

The diagnosis of IgG4‐RD necessitates careful integration of clinical, radiological, and histopathological data. In this case, the characteristic histopathological features, coupled with the patient's clinical presentation and previous inconclusive biopsies, led to the diagnosis of IgG4‐RD [1].

The diagnostic challenge of IgG4‐RD for hematologists is heightened by the overlapping of clinical and laboratory features with those of a number of other hematologic diseases, including lymphoma, plasma cell neoplasms, and histiocyte disorders [2].

IgG4‐RD, CD, and sarcoidosis share overlapping features, such as lymphadenopathy and immune dysregulation. However, they differ significantly in pathophysiology [3], diagnostic markers, and treatment strategies. Differentiating these conditions is critical because of the unique therapeutic implications.

Differential diagnosis can be challenging since CD and IgG4‐related disease (IgG4‐RD) can appear with comparable clinical characteristics, especially in terms of lymphadenopathy and organ involvement. Both disorders can cause swelling of lymph nodes and may affect several organ systems, including the pancreas, retroperitoneum, and salivary glands, which are also often affected in IgG4‐RD.

Differentiating between the two is essential, though, because the treatment approaches are hugely different. Elevated blood IgG4 levels, distinctive histological features (storiform fibrosis and IgG4‐positive plasma cells), and organ‐specific involvement are the hallmarks of IgG4‐RD, an immune‐mediated systemic disease, whereas CD is largely a lymphoproliferative disorder. To distinguish between the two conditions, the biopsy and serum IgG4 tests are essential [4].

Due to its rarity and the fact that its clinical and histological characteristics might overlap with those of other illnesses, CD can be difficult to diagnose. Imaging investigations and the patient's initial appearance pointed to a persistent inflammatory or viral disease.

A benign growth of lymphoid tissue is a characteristic of CD, a rare disorder that can spread to several lymph nodes or be unicentric (UCD), involving only one lymph node. The disorder is divided into three groups: mixed, plasma cell, and vascular hyaline. The most common and usually asymptomatic kind is hyaline‐vascular, which is incidentally identified by imaging and confirmed by postoperative biopsy.

There are two clinical forms: the multicentric form manifests with more severe symptoms, such as fever, weight loss, and widespread lymphadenopathy, and has a more aggressive course than the unicentric form, which is localized and usually less symptomatic. CD is often found by accident during normal screenings or as a result of symptoms of local compression. Although the incidence of CD is unclear and can affect anyone at any age, the literature primarily reports cases in adults, with a slight 60% female predominance [5].

To confirm the diagnosis, lymph node biopsy histopathological analysis is necessary. While multicentric CD usually comprises peripheral adenopathy and may include splenic and hepatic enlargement, unicentric CD is characterized by masses that are often detected in the pelvis and abdomen.

Our patient, who is presenting with right upper thigh swelling, a big fibrolipoma in the right groin, and Multiple enlarged lymph nodes. Thats why we considered it as Multicentric which is misdiagnosed with cellulites, he was admitted multiple times and received several types of antibiotics before being discharged. He has been admitted many times with the same complaint of fever and pain in his leg and was treated as a case of cellulitis, which is what delayed his diagnosis.

Flow cytometry analysis shows Prominent lymphoid hyperplasia within the lymph nodes and soft tissue associated with numerous eosinophils; Sclerosis and fibrosis in the soft tissue confirm the diagnosis.

Despite a confirmed diagnosis, the patient did not receive appropriate treatment due to loss to follow‐up, highlighting the importance of continuous care and patient education in managing chronic diseases.

The 2019 American College of Rheumatology/European League Against Rheumatism (ACR/EULAR) Classification Criteria are the most often used among various diagnostic criteria for IgG4‐Related Disease (IgG4‐RD). There are three steps in the diagnostic technique. First, there must be involvement of at least one of the 11 organs that are frequently affected by IgG4‐RD, including the pancreas, retroperitoneum, lacrimal glands, and salivary glands. The following phase is to apply a set of 32 exclusion criteria, which include clinical, radiologic, pathologic, and serologic variables. The diagnosis of IgG4‐RD is ruled out if any of these exclusion criteria are met. Lastly, eight weighted inclusion criteria that assess radiologic, histological, clinical, and serologic outcomes form a component of the classification. Following these criteria, a score of 20 points or more qualifies as diagnostic for IgG4‐RD [6].

Considering the possibility that the lesions linked to IgG4‐RD might resemble tumors, infections, or other immune‐mediated disorders, patients with this syndrome are sometimes mistaken for having a malignancy.

Clinical, radiographic, serological, and histological characteristics are all combined in the Japanese Comprehensive Clinical Diagnostic(CCD) [7] Criteria to diagnose IgG4‐RD. Particular organ involvement, high IgG4 levels, distinctive imaging characteristics, and histological findings such as the presence of IgG4‐positive plasma cells and fibrosis are all given particular weight in this method. By employing this comprehensive method, medical professionals may correctly identify IgG4‐RD and distinguish it from other diseases that reveal comparable clinical manifestations [7].

While immunological dysregulation is a common feature of autoimmune diseases such as sarcoidosis and IgG4‐RD, their underlying processes differ. Although there are some similarities to IgG4‐RD and sarcoidosis in terms of immune system abnormalities, the differential diagnosis may be autoimmune disorders, which are characterized by autoantibodies attacking the body's tissues. Accurate diagnosis of these disorders and exclusion of other conditions with similar symptoms require clinical evaluation, radiologic imaging, histological analysis, and serologic testing. increased IgG4 levels, histology, and particular diagnostic criteria (such ACR/EULAR) are used to diagnose IgG RD [8].

Corticosteroids (prednisone or prednisolone) are the main treatment for IgG4‐related illness. Usually, the dosage is 30–40 mg/day for 2–4 weeks, then tapered gradually over a few months. Patients may be treated with immunosuppressive drugs such as methotrexate, mycophenolate mofetil, or azathioprine [9] if they do not respond well to steroids or need steroid‐sparing therapy. Another choice for refractory instances is rituximab. Usually, surgery is reserved for complications or diagnostic reasons.

Relapses, however, happen 40%–60% of the time, particularly if treatment is not continued. Long‐term monitoring is necessary to address problems and relapses, as chronic organ damage may continue. The prognosis is usually favorable with prompt and effective treatment, but neglected instances may lead to progressive organ damage [10].

However, the prognosis for multicentric CD is often unpredictable, and there is no established consensus on treatment. Combinations of chemotherapy, steroids, and surgical excision have been explored in a number of diverse ways. There is no danger of malignant degeneration from the illness, and the overall prognosis is exceptionally good.

The repeated misdiagnoses and frequent antibiotic courses in this patient demonstrate the challenges of distinguishing inflammatory and infectious conditions from fibro‐inflammatory diseases like IgG4‐RD or lymphoproliferative disorders. This underscores the need for early biopsy and multidisciplinary evaluation in cases of persistent, unexplained lymphadenopathy.

## 4 Conclusion

IgG4‐related disease poses a diagnostic challenge due to its diverse clinical manifestations and histopathological resemblance to other conditions. CD, though uncommon, should be included in the differential diagnosis for patients with chronic, unexplained lymphadenopathy. The rare occurrence of this case report is the reason it is being presented. Although lymphoma and nodal secondaries are two other common causes of inguinal lymphadenopathy, multiple enlarged lymph nodes are implicated in CD. Histopathological examinations are vital for accurate diagnosis, and timely biopsy is crucial for proper management. The preferred diagnostic and therapeutic procedure for unicentric localized type CD is surgical excision of the tumors. This case highlights the importance of healthcare providers considering CD in their differential diagnoses and ensuring patient adherence to scheduled diagnostic procedures. Increased awareness and careful evaluation are essential for achieving prompt diagnosis and initiating appropriate treatment to prevent disease progression and minimize the risk of organ damage. It is imperative that we never overlook the significance of routine follow‐ups to monitor the risk of recurrence, or the fact that every patient diagnosed with CD should get a systemic survey to rule out the chance of overlooked lesions or multiple diseases.

## Author Contributions

**Mohamed Gaafar Mohamedali:** supervision, writing – original draft, writing – review and editing. **Bassem Alhariri:** supervision, writing – original draft, writing – review and editing. **Sana Mohamed:** writing – original draft, writing – review and editing. **Osman Osama Elhassan:** writing – original draft, writing – review and editing. **Muayad Ahmad:** supervision. **Afraa Fadul:** resources, supervision.

## Ethics Statement

This patient provided oral and signed written consent to use his clinical materials in this study. The study was conducted in accordance with the principles of the institutional ethical standards and national research committees.

## Consent

Written informed consent was obtained from the patient to publish this case report and any accompanying images.

## Conflicts of Interest

The authors declare no conflicts of interest.

## Data Availability

The data that support the findings of this study are available in this article.
